# Shall Our Young Men Study the Classics?

**Published:** 1886-01

**Authors:** J. Donald Wilson

**Affiliations:** Professor of Chemistry, Atlanta, GA.


VOL. II. JANUARY, 1886. NO. 11.

### Original Communications.

### SHALL OUR YOUNG MEN STUDY THE CLASSICS?

AN ADDRESS DELIVERED AT THE OPENING OF THE ATLANTA MEDICAL COLLEGE, OCTOBER 7, 1885, BY J. DONALD WILSON, M. D., PROFESSOR OF CHEMISTRY, ATLANTA, GA.

The men who at any time in the world's history possess the most robust thinking power—men of great influence, who mould the thought of their time and control more or less other destinies than their own—may be divided into two classes. The one class expends its powers in carrying on the world's affair, in political strife, great industrial enterprises, colonizing schemes, diplomatic achievements, fighting great battles; men of masterful activity, who are never quiet, who do their thinking on the run, whose thought is no sooner a thought than it becomes a deed, who act history rather than record it, men who spend their energies amid the stir and conflict of events.

The other class, equally influential, is made up of men who do their thinking in a quieter way ; men who s't still, but whose minds do not sit still; men who think and record the result so that others may think; men of the cloister, of the midnight study, of the sanctum, of the laboratory; men whose expressed thought is not a great battle or trans-continental railroad, but a great history, critique, poem, drama, philosophic generalization, scientific treatise or great discovery; men whose work is noiseless, but none the less influential, for they wield that instrument which Lord Lytton has told us "is mightier than the sword."

Passing by the first or active class as not germane to my purpose, and coming to what I have termed the quieter class, this great body of men, whose influence is so potent and far-reaching—who give a direction to the thought of their time and determine, so to speak, the *complexion* of their age—I wish to ask, what are the special characteristics of these men in our times? In what direction is their most telling work accomplished? What kind of thoughts are being elaborated—what kind of work is being done which will especially distinguish our time from any other which has preceded it?

No one who knows the facts will deny that there is a sad degeneracy in purely literary work. Our literary men are mere pigmies compared with those who have gone before. Selecting carefully six of our greatest men of letters—if, indeed, you can find as many worth the naming—how does their work compare with that of the Elizabethan era? It is "as moonlight unto sunlight, and as water unto wine." We do not excel in oratory, nor poetry, nor painting, nor æsthetics, nor architecture, nor in imaginative and dramatic writing. Hardly a drama worth reading has appeared in many a decade. In all of these we occupy the position of passable mediocrity. What then is the genius of our time? What is the special bent of the greatest minds in the higher realms of mental activity? The question answers itself: Science, scientific investigation, scientific thinking, scientific discovery. It goes without saying that this is the one great predominating characteristic by which our age lays claim to distinction. Ours, far more than any other age which has preceded it, is imbued with scientific ideas. Not only do we know more about Nature and Nature's methods through the myriads of discoveries and the careful elaboration of the various sciences, but the *Spirit* has become contagious, and we find all classes of subjects handled after the scientific method.

Now it would be strange indeed if these facts could obtain without producing striking and fundamental changes in the world of thought. However much any Bourbon may deny it, it has affected most profoundly in manifold directions the tenor of human inquiry. For instance, this is manifest in the changed character of the religious discussion. This has shifted into a different sphere of thought. In former days he had the best of it who could quote the most authorities. Learning was held at a higher premium than thinking. "It is written" settled the dispute. But in with this modern tide comes a constantly increasing disrespect for authority. We are growing jealously skeptical as to whether men who lived ages ago, and who knew so much less of Nature and her methods than we do, have a right to control the direction of our thoughts. Let us reason together, but let us have our facts at first hand. Let us philosophize (all men must philosophize), but let us examine these questions in our own way, and with our own implements, throwing off the incubus and the *ipse dixit* of antiquity. Such is the spirit that is abroad in the world. Remember, I have nothing to say on one side or other of this question. That is not my business. I mention this simply as an illustration of the fact that this scientific tendency has changed from their very foundations some of the standpoints and landmarks in questions of vast import to all the thinking sons of men. It is in the direction of another innovation that I would direct your ideas. This new generation of thinkers has dared to rise up in revolt against the system of education which has descended to us from our fathers. They have the impudence and the audacity to tell us that the system now in vogue, which is not the product of our day at all, but of mediæval times, is a crippling inheritance which impedes our progress, and they call upon us to effect our emancipation. They tell us we ought not to build a school-house with all modern improvements and teach in it what was taught in the middle ages. Now when the spirit of our time speaks to us, shall we not listen to its voice?

If we would be in sympathy with the genius of our age, we should take heed when its representatives speak, and I know of no better way to come straight to the point than to take as a text some sentences from one of this newer class of thinkers. He says:

"Among mental, as among bodily acquisitions, the ornamental comes before the useful. Not only in times past, but almost as much in our own era, that knowledge which conduces to personal well-being has been postponed to that which brings applause. We are guilty of something like a platitude when we say that throughout his after career a boy, in nine cases out of ten, applies his Latin and Greek to no practical purpose. Generally the greater part of it drops out of his memory, and if he occasionally vents a Latin quotation, it is less to throw light on the topic in hand than for the sake of effect. If we inquire what is the real motive for giving boys a classical education, we find it to be simply conformity to public opinion. Men dress their children's minds, as they do their bodies, in the prevailing fashion. As the Orinoco Indian puts on his paint before leaving his hut, not with a view to any direct benefit, but because he would be ashamed to be seen without it, so a boy's drilling in Latin and Greek is insisted on, not because of their intrinsic value, but that he may not be disgraced by being found ignorant of them—that he may have the education of a gentleman. In the attiring of women the desire of approbation overrides the desire for warmth and convenience, and similarly in their education, the immense preponderance of 'accomplishments' proves how here, too, use is subordinated to display."

These, to my thought, are words of very great weight.

It is contended by men of the old regime that our boys, during the formative or plastic stage of their minds, ought to read the great master-pieces of Greece and Rome, because this gives them a literary and humanitarian culture which they cannot get in any other way. At first blush this seems plausible enough. But let us examine the facts. Let us consider the matter among American students, not as it ought to be, nor as it is pretended to be, but as it really is.

Can a boy get literary culture out of something which he does not at all appreciate *as literature?* Something which he reads simply as a difficult task? Something in which he generally sees no beauty, and from which he derives no pleasure? In the actual practical working of the system, not five in a hundred ever get sufficient grip on either of these languages to have command of their literature. Can a boy appreciate the rhythm and movement of a Greek poem when he worries out the meaning of the lines with the most excruciating difficulty? Can you get culture from a language when you cannot direct your thought to the ideas, but must have your attention continually distracted by painful search for the meaning of words? Is it the words we are after? Perhaps I made a mistake. I thought it was the ideas. I always thought ideas were more valuable than words, both for purposes of culture and for every other purpose. Our young men study Latin and Greek for a long number of years, and yet at graduation are unable, *in any true sense*, to read either one. Does all this pretended culture come to us from a language which we cannot read? Is it right to spend time and money in learning *anything* as imperfectly as most of our boys learn Latin and Greek? Is it not an actual training in doing things by halves and in a smattering way? Does it not conduce to slovenly mental habits? And then what is it they are studying so faultily? They spend this valuable time in getting the merest smatter of the literature of nations long ago dead and in studying events which have not now any vital or controlling interest in human affairs, and they are studying even this in the most imperfect and superficial way.

Philip Gilbert Hamerton tells of a certain Frenchman whom he met, who had acquired an acquaintance with English, which would in the case of a dead language be considered a very high degree of excellence. He knew most of our best literary work even down to a close comparison of different readings. He had gathered together such stores of information in English literature that few educated Englishmen could compare with him, but he could not talk or write the language in a way that was not laughable to a native born. He knew nearly all the words in our language, but it was dictionary knowledge, and it differed so from a native's apprehension of the same words that his English was a sort of false English, and not our living tongue at all. He differed so very widely from English criticism and English feeling in his opinions of our various authors that he evidently did not understand them as we understand them. He could not tell the difference between declamatory verses and bombastic oratory and work of a really good quality, and often mistook the one for the other. Now this man had just the same kind of knowledge of English that our young men get of Latin and Greek, with this difference, that the majority of our students never arrive at a tenth part of the excellence in Latin and Greek that he had arrived at in English. And yet we are told they are getting a vast deal of literary culture out of it. "Something is rotten in the State of Denmark." I believe an occasional scholar like Lord Macaulay does gain a really valuable knowledge of the classics, but shall we found our educational system on what the rare exception can do? No community should be taxed to support a system of education which fails with the ninety and nine and succeeds only with the occasional scholar who, from his birth, is endowed with literary instincts.

Again, we are told that the study of the classics is a valuable aid in acquiring a good command of English. The study of English literature will show that we have just as good writing in any or all directions from those ignorant of the classics as from those learned in them. Shakespeare was an instance of this, for he knew, as his scanty biography tells us, little Latin and less Greek. This of itself would show that they are not indispensable. I can never believe that the study of the speeches of Greek and Roman lawyers, which have no practical interest for us, and in the imperfect way which I have mentioned, can ever equal for this purpose the study of the great speeches of the best English lawyers and statesmen. It is by the constant use and study of our own language that we become adepts in handling it. No one would think of taking as models of good English articles from college journalism. They are stilted and unnatural in the extreme, and yet they are produced while the boys are in closest contact with Latin and Greek. They tell us that the Greeks had the most perfect language the world has yet seen. How was it produced? The Greeks never studied any language but their own. Terse and vigorous English is the result, more than anything else, of close and vigorous thinking, and this is not produced by studying any language, but by studying something else entirely.

I would not be understood as arguing for a purely scientific education as opposed to the literary, not at all. Let us give our children literary culture, but let it be done by the study of something which will really give it to them.

When the friends of the classical system, in their conflict with the modern idea, have from time to time been hard pushed, they have shifted their ground, and so we find the gymnastic idea advanced. They tell us a boy enters college for the training of his mental powers, just as an athlete enters the gymnasium for the training of his physical powers, and it is as absurd to complain that the boy does not carry all of his Latin and Greek away from college as it would be for the athlete to complain that he could not carry away the dumb bells and Indian clubs from the gymnasium. He goes there, not so much for the intrinsic value of what he learns, but for development and discipline.

Now my objection to this is that the majority of mankind cannot afford to learn that which is not valuable in itself simply for the discipline. Admitting that discipline is the all-important thing, the modern idea is, can we not get the discipline by the study of something which we can use in our daily life, and thus have two results instead of one? And to this end we wish to give the sciences—the close and analytical study of nature—an integral and predominating place in early education.

Most of us are not rich enough to go to school for the training of our minds alone. Our education must be, in some sense, a preparation for our work in life, and we feel that we ought to get the ed-u-ca-tion—the *e* and the *duco*—the leading out of our minds by the study of facts which will serve us in our daily conflicts. Suppose the classics are excellent as discipline, what kind of discipline is it? If it be anything, it must be literary discipline.

How many of us are going to spend our lives in literature? Most of us are dealing with things and facts, not with words. "To study expression before subjects of thought have been accumulated is to cultivate the habit of talking fluently without having anything to say."

In what way does the study of the classics discipline the mind? Is it not almost wholly the memory? This, of course, must be cultivated—it is of great importance, but shall we spend so much time in cultivating that which is not the highest faculty of the mind to the neglect of the reason and judgment which are so much more important?

We are told that mathematics has a large space in the curriculum, and that this cultivates the reasoning faculty so that the mind is developed symmetrically. Mathematics is a valuable study, chiefly for the reason that it trains us to concentrate the attention and follow continuously one line of thought; but Sir William Hamilton long ago pointed out the fact that this study teaches us to reason only in quantities, and that the mind, long accustomed to this kind of drill, becomes unfit to take the qualities of things into consideration, and yet quality is of prime importance in all other kinds of reasoning. Can you not all call to mind some man, brilliant, perhaps, in mathematics, but lame, narrow and bigoted when it comes to reasoning about other matters? In mathematics we start to reason from premises which are granted. We begin with given points, which everybody admits. How is it in our reasoning about daily affairs? The chief difficulty is to gather the proper facts, get the correct data, form the premises aright and then the deductions are easy. As a training for this kind of work, nothing can compare with the sciences.

It is nature's method to give the child a desire to pry into and examine everything which surrounds it in the world. Here is a golden opportunity to impress the relations of cause and effect upon its mind, to start the infant intellect in processes of reasoning, and yet we know that this propensity is more often repressed than encouraged. A child which studies language almost exclusively forms a habit of admitting exceptions to the rules of grammar, and is apt to think there are exceptions to other laws, physical and moral. It does not have impressed upon its mind the necessity, the inevitableness of the laws under which we do all of our work in the world. What would we think of the man who, when the window fell upon his child, began to pray God to spare its life before he lifted the window? Then, what shall we say of those good people, who, when a great scourge visits their city, betake themselves to prayer before their streets are thoroughly cleansed? Being unable to see the disease germs, they attribute their trouble to some mysterious visitation of Providence.

It is not enough that one should study nature through one of the sciences later in life. The study of natural phenomena should begin when the mind is in the bud. Our system of education should especially exercise the powers of observation, comparison, analysis, judgment and reasoning when the mind is in its plastic stage, so that a logical habit of thought will be engendered which will cling to one through life.

I once heard a lecture from a teacher of science upon the law of the diffusion of gases. After telling his audience that the oxygen and nitrogen of the atmosphere did not separate into different layers, the one being above and the other below, but, by virtue of this law, were equally diffused throughout each other, he told us how thankful we should be to our Creator that such a law existed, for, said he, if the oxygen all remained below, we would breathe so fast we would soon burn up, or if the nitrogen all remained below we would at once choke. I have many things to be thankful for, but I shall never so stultify myself as to be thankful that I was not strangled to death the moment I was born. The Creator has seen fit to bring living beings into the world. Would it not have been strange if he had made such laws that his creatures could not exist at all? And yet I hear a great deal of just such reasoning from the pulpit. Our Sunday-school books, which are great educators of the young, are filled with things which give the growing minds of our children exceedingly illogical and unfortunate modes of thought.

This teacher I speak of had read the classics a great deal, and was an excellent mathematician. Later in life he had studied the sciences, but he had not studied them during the formative period of his mind. If our legislators had been educated after the method for which I plead, our anatomical bill would have passed long ago; but many of them have not that in their minds upon which an intelligent physician can work to begin the process of conviction. In spite of the contempt which the classicists heap upon a practical education, is it not a pity that any child's eye should be permanently ruined by cross-eyes because the parents do not know that it can be remedied? Is it not a pity that any child should die of the acid which it finds upon the mantel-piece, when, in the cupboard, within a few feet of the poor mother, are always to be found alkalies? It seems to me specially needful that we should have physiology and hygiene thoroughly taught in the public schools. The hope of the medical philosopher for the future lies in the direction of preventive medicine. But sanitarians are powerless without the hearty co-operation of the people, and this we cannot hope for until we have a better instructed public opinion.

In conclusion, I would say to you young men who have come here to pursue a course of studies, endeavor from the beginning to cultivate habits of analysis and reasoning rather than that of memorizing; but if you find, as you surely will find, that part of your work must be done by the memory, let this part of it be done with definiteness and exactitude. Do not fall into slovenly habits of any kind. And if you ask me for a motto to write over your door, now in the beginning of your career as students, take these words of Confucius: "What we know, to know that we know it; what we do not know, to know that we do not know it; this is knowledge."

